# Dynamic Remodeling of Individual Nucleosomes Across a Eukaryotic Genome in Response to Transcriptional Perturbation

**DOI:** 10.1371/journal.pbio.0060065

**Published:** 2008-03-18

**Authors:** Sushma Shivaswamy, Akshay Bhinge, Yongjun Zhao, Steven Jones, Martin Hirst, Vishwanath R Iyer

**Affiliations:** 1 Institute for Cellular and Molecular Biology, Center for Systems and Synthetic Biology, and Section of Molecular Genetics and Microbiology, University of Texas at Austin, Austin, Texas, United States of America; 2 Michael Smith Genome Sciences Center, British Columbia Cancer Agency, Vancouver, British Columbia, Canada; University of Massachusetts Medical School, United States of America

## Abstract

The eukaryotic genome is packaged as chromatin with nucleosomes comprising its basic structural unit, but the detailed structure of chromatin and its dynamic remodeling in terms of individual nucleosome positions has not been completely defined experimentally for any genome. We used ultra-high–throughput sequencing to map the remodeling of individual nucleosomes throughout the yeast genome before and after a physiological perturbation that causes genome-wide transcriptional changes. Nearly 80% of the genome is covered by positioned nucleosomes occurring in a limited number of stereotypical patterns in relation to transcribed regions and transcription factor binding sites. Chromatin remodeling in response to physiological perturbation was typically associated with the eviction, appearance, or repositioning of one or two nucleosomes in the promoter, rather than broader region-wide changes. Dynamic nucleosome remodeling tends to increase the accessibility of binding sites for transcription factors that mediate transcriptional changes. However, specific nucleosomal rearrangements were also evident at promoters even when there was no apparent transcriptional change, indicating that there is no simple, globally applicable relationship between chromatin remodeling and transcriptional activity. Our study provides a detailed, high-resolution, dynamic map of single-nucleosome remodeling across the yeast genome and its relation to global transcriptional changes.

## Author Summary

The eukaryotic genome is packed in a systematic hierarchy to accommodate it within the confines of the cell's nucleus. This packing, however, presents an impediment to the transcription machinery when it must access genomic DNA to regulate gene expression. A fundamental aspect of genome packing is the spooling of DNA around nucleosomes—structures formed from histone proteins—which must be dislodged during transcription. In this study, we identified all the nucleosome displacements associated with a physiological perturbation causing genome-wide transcriptional changes in the eukaryote Saccharomyces cerevisiae. We isolated nucleosomal DNA before and after subjecting cells to heat shock, then identified the ends of these DNA fragments and, thereby, the location of nucleosomes along the genome, using ultra-high–throughput sequencing. We identified localized patterns of nucleosome displacement at gene promoters in response to heat shock, and found that nucleosome eviction was generally associated with activation and their appearance with gene repression. Nucleosome remodeling generally improved the accessibility of DNA to transcriptional regulators mediating the response to stresses like heat shock. However, not all nucleosomal remodeling was associated with transcriptional changes, indicating that the relationship between nucleosome repositioning and transcriptional activity is not merely a reflection of competing access to DNA.

## Abstract 3

Ultra-high-throughput sequencing is used to show that distinct, localized patterns of nucleosome repositioning at promoters underlie the genome-wide transcriptional response to a physiological stimulus.

## Introduction

The eukaryotic genome is compacted into nucleosomal arrays composed of 146-bp DNA wrapped around a core histone octamer complex [1]. The location of nucleosomes affects nearly every cellular process requiring access to genomic DNA, but it is not well understood how nucleosomes are positioned and remodeled throughout any genome. Mapping nucleosome positions using DNA microarrays covering 4% of the yeast genome has shown that a majority of assayable nucleosomes were well positioned [2]. Computational analyses incorporating structural mechanics of nucleosome associated DNA [3–5] and comparative genetics [6] have predicted nucleosome positions in the yeast genome. However, experimental validation and comparison with available in vivo data show that intrinsic signals in genomic DNA determine only 15%–17% of nucleosome positioning above what is expected by chance [3,4]. In vivo nucleosome positions are influenced by the presence of numerous ATP-dependent remodelers, and the transcriptional machinery [7,8].

Recently, chromatin immunoprecipitation (ChIP)-sequencing technology was used to map the positions of nucleosomes containing the variant H2A.Z histone across the yeast genome [9]. H2A.Z nucleosomes are enriched at promoters; therefore, this study mapped about 10,000 nucleosomes. Tiling arrays have been recently used to catalog the positions of nucleosomes at 4–5-bp resolution across the yeast genome and their repositioning by chromatin remodelers [10,11]. However, dynamic changes in individual nucleosome positions in response to physiological perturbations that cause global transcriptional reprogramming have not yet been examined on a genomic scale in any organism.

To map the location of individual nucleosomes on a genomic scale and at high resolution, we used ultra-high–throughput sequencing methodology (Solexa/Illumina) to sequence the ends of nucleosome-associated DNA. Our approach enabled us to map individual nucleosomes nominally at single-nucleotide resolution. Nucleosome density and stability at promoters and over coding regions were correlated specifically with transcription rate rather than absolute transcript levels. Two different modes of chromatin remodeling were associated with transcriptional regulation. Gene activation was mainly accompanied by the eviction of one to two nucleosomes from the promoter, and gene repression was mainly accompanied by the appearance of nucleosomes with varying stability over the promoter. Our work constitutes the first study of dynamic single-nucleosome remodeling in response to transcriptional perturbation across an entire eukaryotic genome.

## Results

### Strategy for Identifying Nucleosome Positions Using Ultra-High–Throughput Sequencing

We used micrococcal nuclease to isolate mononucleosome-associated DNA from yeast cells before and after a physiological perturbation (heat shock for 15 min) that causes genome-wide transcriptional changes, and sequenced the ends of the fragments. Only uniquely aligning reads were used to define the ends of nucleosomal DNA. After aligning sequence reads to the genome, we defined nucleosome peaks by first using a Parzen window probability estimation of read densities, then defining a peak of width 146 bp around the centers of appropriately spaced maxima in the density function (Materials and Methods). Our approach yielded nucleosome positions at single-nucleotide resolution. We calculated a score for the position and stability of each nucleosome, which were normalized to account for differences in sequencing depth. Scores in the range of 0.2 to 0.25 and higher indicated nucleosomes whose positions often matched in the two independent biological samples and, hence, indicated bona fide nucleosomes; nucleosomes below this threshold were defined by too few reads to be discernable above background. At a score cutoff of 0.25, we defined the locations of 49,043 nucleosomes in normally growing cells and 52,817 nucleosomes in heat-shocked cells. Assuming that two adjacent nucleosomes cannot be closer than 200 bp, altogether about 73% of the yeast genome is covered by a positioned nucleosome. Since only uniquely aligning reads were used in our analysis, and the yeast genome contains an appreciable fraction of repeated sequence elements, we estimate that about 78% of the genome is covered by positioned nucleosomes.

### Recapitulation of Known Nucleosome Positions and Expected Remodeling Events

**Figure 1:**
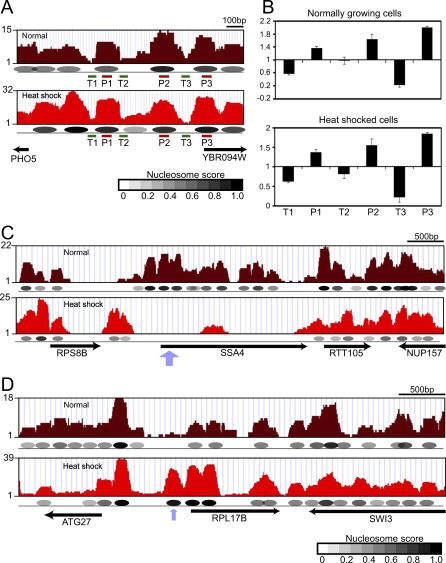
Ultra-High–Throughput Sequencing Recapitulates In Vivo Nucleosome Positions (A) Detailed view of the *PHO5* locus showing the raw sequence reads (brown and red profiles). The nucleosome positions calculated using our analysis algorithm are shown as ovals, shaded according to their nucleosome score as indicated. The positions of the amplicons used for qPCR analysis are marked as red (peaks) and green (troughs) lines below. The black arrows indicate the positions of genes in that region. (B) qPCR verification of the three nucleosome peaks and three troughs identified by sequencing confirm that their positions remain the same before and after heat shock. (C) The heat-shock–induced SSA4 gene and flanking regions, showing that nucleosomes are displaced specifically at the SSA4 promoter and coding region after heat shock (thick purple arrow). (D) The heat-shock–repressed ribosomal protein gene *RPL17B* and flanking regions, showing that a single positioned nucleosome appears after heat shock specifically at the RPL17B promoter (thin purple arrow). The nucleosome positions calculated using our analysis algorithm are indicated as in (A).

We assessed the quality and accuracy of our nucleosome sequencing data by examining the nucleosomes known to be positioned at the *PHO5* promoter. The yeast *PHO5* promoter is repressed during growth in rich media by specifically positioned nucleosomes flanking a short, hypersensitive region containing a binding site for the transcription factor Pho4 [12]. These nucleosomes were evident in the alignment of our raw sequence reads, and their precise positions calculated by our analysis algorithm corresponded to the known positions of these nucleosomes. The positions of these three nucleosomes did not vary in the two independent biological samples before and after heat shock, as this perturbation does not affect the *PHO5* promoter (Figure 1A). Quantitative real-time PCR (qPCR) for the three nucleosome peaks and three troughs (linker regions) identified by sequencing provided independent experimental verification of these nucleosome positions and the fact that their positions did not change in the two samples (Figure 1B). At individual promoters where transcription is activated by heat shock, the raw data traces and our inferred nucleosome peaks showed that nucleosomes were displaced at the promoter after the perturbation (Figure 1C). Conversely, at promoters that are repressed, positioned nucleosomes appeared after the perturbation (Figure 1D). The genome-wide nucleosome positions we identified experimentally correspond well with individual nucleosomes mapped on chromosome III as well as nucleosome-bound sequences isolated in previous studies [2,4] (see Figure S1 and Table S1). While this manuscript was in preparation, a catalog of nucleosome positions in yeast was published [13]. Our mapped nucleosome positions also agree well with this recent study (Figure S1 and Table S1). Thus, our mononucleosome preparations and the high-throughput sequencing assay recapitulated bona fide in vivo nucleosome positions and rearrangements.

### Lower Nucleosome Occupancy at Promoters Compared to Coding Regions

**Figure 2:**
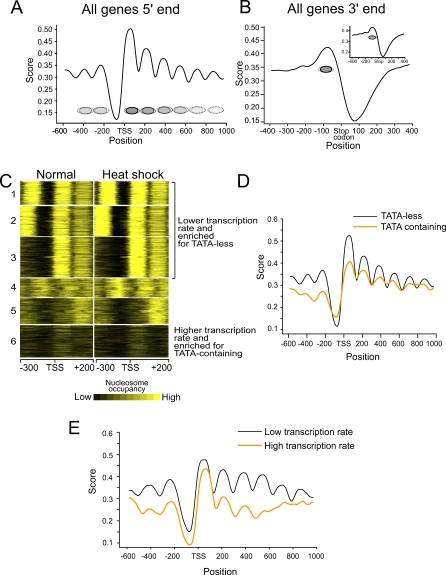
Patterns of Chromatin Organization in the Yeast Genome (A) Average nucleosome profiles of all genes in the yeast genome from −600 bp to +1,000 bp with respect to the transcription start site (TSS). Nucleosome positions are shown as gray ovals below the profile. The intensity of the filled oval reflects the average probability score of the nucleosomes (see Figure 1 for the color scale), and the dotted oval marks the spread of that nucleosome across all genes. (B) The 3′ end of genes is marked by a strongly positioned nucleosome, followed by a relatively nucleosome-free region. The inset shows the 3′ end of convergently transcribed genes in which the 3′ end is not followed by another promoter. (C) Distinct classes of nucleosome profiles revealed by *k*-means clustering of all promoters in the yeast genome. Each row in the clusters shows the position of a nucleosome at an individual promoter. Nucleosomes are colored according to their probability using the shown color scale. Clusters 1–3 showed a significant enrichment for genes with lower transcription rates and for TATA-less genes (*p* ≤ 10^−10^). Cluster 6 showed a significant enrichment for genes with high transcription rates and for TATA-containing genes (*p* < 10^−10^). (D) Average nucleosome profiles for TATA-containing (973) and TATA-less (4,382) promoters, aligned with respect to the TSS. (E) The genes in the yeast genome were sorted in descending order according to their transcription rates [16], and the average promoter nucleosome profiles for the top 500 genes (orange) and the bottom 500 genes (black) are plotted.

Low-resolution analysis using PCR microarrays has shown that promoters are nucleosome-poor relative to coding regions [14,15]. In accord with these findings, we found that both the number and the stability of nucleosomes were significantly lower at promoters than over coding regions (*p* < 2.2 × 10^−16^). We plotted the average nucleosome profile over all yeast genes to get an idea of how individual nucleosomes were distributed in relation to promoters and coding regions. Several features of chromatin organization were evident from this plot (Figure 2A). First, as noted before, promoters showed a lower probability of nucleosomes as compared to coding sequences. Second, the apparent nucleosome-free region immediately upstream of the transcription start site (TSS) is only approximately the width of a single nucleosome. Third, there is a strongly positioned nucleosome, likely an H2A.Z-containing nucleosome, that marks the start of the transcribed region immediately downstream of the TSS [9]. Fourth, positioned nucleosomes continue at periodic intervals downstream of the TSS, with decreasing probabilities. These characteristics of nucleosome positioning corroborate results based on mapping nucleosomes across a single yeast chromosome [2]. We obtained nearly identical results in the independent heat-shocked cells (Figure S2). Interestingly, we also observed a strongly positioned nucleosome at the 3′ end of the coding region followed by a relatively nucleosome-free region, which has not been noted before. This 3′ nucleosomal mark does not reflect the boundary of a downstream promoter, because it was evident even at the 3′ end of convergently transcribed genes lacking another promoter immediately downstream of their 3′ end (Figures 2B and S2). This 3′ end chromatin feature was not biased towards convergently transcribed genes, but we noted a modest association with genes that were expressed at low levels and with long genes (unpublished data). Our data also established that although the internucleosomal linker length could vary widely, the linker length is commonly about 30 bp in the yeast genome (Figure S3).

### Nucleosome Positioning Is Influenced by the Presence of a TATA Box and Is Correlated with Transcription Rate

Although our whole-genome data revealed stereotypical distribution patterns of nucleosomes around promoters, we reasoned that the average profile might conceal several distinct nucleosome occupancy profiles with distinct relationships to transcriptional activity or promoter sequence characteristics. To reveal such distinctions, we performed *k*-means clustering of the nucleosome peak profiles around all yeast promoters. Indeed, several classes of nucleosome profiles were now evident (Figure 2C). There was no significant distinction between these different promoter classes with respect to either their occupancy by the general transcription factor TBP or their absolute transcript levels (Figure S4). However, there were biases among the clusters with respect to their representation of TATA box–containing and TATA-less promoters, as well as their transcription rates. In general, promoter classes containing a strongly positioned nucleosome were enriched for TATA-less promoters and had lower transcription rates, and conversely, the cluster containing poorly positioned nucleosomes was enriched for TATA-containing promoters and had higher transcription rates [16] (Figure 2C). We ascertained that promoters that appeared to be largely devoid of positioned nucleosomes were not artificially caused by our exclusion of ambiguous sequence reads. The average nucleosome occupancy profiles for TATA-less and TATA-containing promoters, considered separately, showed that the absence of a consensus TATA element in the promoter was indeed correlated with the stereotypical genome-wide nucleosome profile (Figures 2D and S2). This distinction was not due to the lower number of TATA-containing promoters (unpublished data). Correspondingly, genes with low transcription rates showed stronger nucleosome positioning as compared to genes with higher transcription rates (Figure 2E).

**Figure 3:**
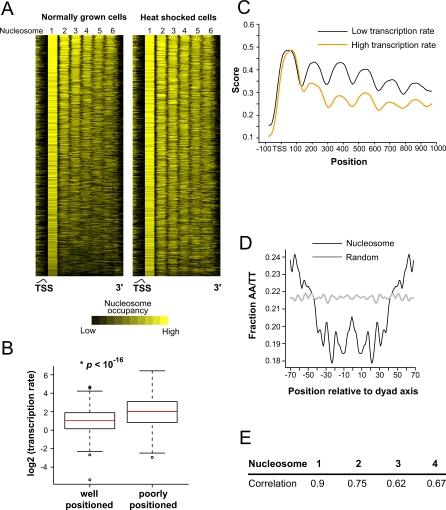
Nucleosome Positioning over Coding Regions Depends on Transcription Rate and Sequence Characteristics (A) Genes were aligned to the first nucleosome downstream of the TSS and sorted by their nucleosome positioning periodicity (NPP) score (see Materials and Methods). Genes were sorted by their NPP scores in normally growing cells, and the data from heat-shocked cells are shown in the same order. The unaligned TSS is indicated by the approximate curve. (B) The transcription rate of genes with high NPP scores (well-positioned nucleosomes) is significantly lower than that of genes with low NPP scores (poorly positioned). In these box plots, the red line indicates the median, the upper and lower bounds of the box indicate the interquartile range, the horizontal lines that are connected to the box by a dashed line indicate the upper and lower bounds of nonoutlier values, and the open circles indicate outliers. (C) Genes were sorted in descending order according to their transcription rates, and the average nucleosome profiles over the coding regions for top 500 genes (orange) and the bottom 500 genes (black) are plotted. (D) Frequency of AA/TT dinucleotide at each position in the DNA sequence associated with the most strongly positioned first nucleosomes. The frequency profiles for the dinucleotides AA and TT for the first nucleosome shown in (A) were summed and smoothed using a 3-bp moving average. The same analysis was also performed for a comparable set of randomly chosen DNA sequences from the yeast genome. (E) Correlation coefficients of the AA/TT profiles for the DNA sequences underlying each of the indicated coding nucleosome positions from (A), with the positioning profile derived earlier. Each of the correlation values was significantly higher than background.

Visual inspection of nucleosome profiles before and after heat shock indicated that the positions of the majority of nucleosomes were closely maintained despite the genome-wide transcriptional perturbation (Figure S1). In general, individual nucleosome positions in each of the promoter classes were largely unchanged in cells after heat shock (Figure 2C). Approximately 65% of all positioned nucleosomes throughout the genome in normally growing cells were within 30 bp of their positions in heat-shocked cells. At a score cutoff of 0.25, less than 10% of the nucleosomes were displaced more than 100 bp after heat shock (Table S1). In addition to the promoter nucleosome classes, we also observed strong, periodically positioned nucleosomes located over the transcribed regions of most genes in the genome. This periodicity was evident when we aligned all coding regions to the first nucleosome downstream of the TSS and ranked all these genes by a nucleosome positioning periodicity (NPP) score applied to the coding region (Figure 3A; Materials and Methods). There was no correlation between NPP and steady-state transcript levels (unpublished data). However, genes with a high NPP score, which had strongly positioned nucleosomes over the coding region, were transcribed at significantly lower rates than genes with a low NPP score (Figure 3B). Correspondingly, genes that were transcribed at low rates showed well-positioned periodic nucleosomes over the coding region relative to genes transcribed at higher rates, which showed weaker nucleosome positioning over the coding region (Figure 3C). Overall, the stereotypical positioning of nucleosomes over coding regions and promoters is consistent with the notion that nucleosome positions in the yeast genome are not random, but rather, are strongly encoded intrinsically through a combination of DNA sequence composition and binding of other proteins.

### Sequence-Dependent Positioning of Nucleosomes

Analysis of DNA sequences associated with nucleosomes has indicated that nucleosome positions are intrinsically encoded in DNA [4,6,13]. However, it is not clear to what extent DNA sequence governs nucleosome positions compared to other factors that might also contribute to nucleosome positioning across the genome. One possibility is that when a nucleosome is strongly positioned at one site by virtue of DNA sequence, immediately adjacent nucleosomes are “stacked” against it and therefore show little sequence dependence. In particular, the regular array of nucleosomes we observed over coding regions could reflect sequence-dependent positioning of an H2A.Z nucleosome at the 5′ end of the array corresponding to the TSS, but with the remainder being positioned relative to the first one in a sequence-independent manner. To test this idea, we examined the sequence dependence of successive nucleosome positions in the strongly positioned nucleosomal arrays over the coding region. We first generated a profile of the AA/TT dinucleotide frequency for the sequences associated with the strongest positioned nucleosomes at the first position shown in Figure 3A. Like the profile generated from computational predictions of nucleosome positions [4,6], our profile shows a repeating pattern with an approximate periodicity of ten nucleotides, indicative of the rotational positioning of the nucleosome over a preferred sequence (Figure 3D). Although the information content of our measured dinucleotide profile is modest, it is significantly different from the same dinucleotide profile measured over randomly selected DNA sequences from the genome (Figure 3D). We then measured the average correlation between our nucleosome sequence profile and the same dinucleotide profile for the set of sequences associated with all nucleosomes in each of the positions in the regular array of coding region nucleosomes. As expected, the first position showed the strongest correlation to the positioning sequence, but in general, successive nucleosome positions in the arrays showed lower, but significant, correlations to the positioning DNA profile (Figure 3E). Thus, although the underlying DNA sequence as measured by the dinucleotide profile makes only a modest contribution to the positioning of nucleosomes, in general this contribution is maintained to a large extent even when nucleosomes are adjacent to another well-positioned nucleosome in the coding region.

### Nucleosome Remodeling Is Mechanistically Linked to Dynamic Changes in Transcription

In order to examine how dynamic remodeling of individual nucleosomes was globally related to dynamic changes in transcription after the physiological perturbation, we generated nucleosome remodeling profiles for all promoters (Materials and Methods). A positive value in the remodeling profile at a given promoter position indicated that there was a nucleosome covering the position during normal growth, but was depleted or evicted upon heat shock. A negative value indicated the opposite, namely, the appearance of a more strongly positioned nucleosome following heat shock. We grouped the remodeling profiles by *k*-means clustering and visualized specific patterns of nucleosomal changes at the promoter.

**Figure 4:**
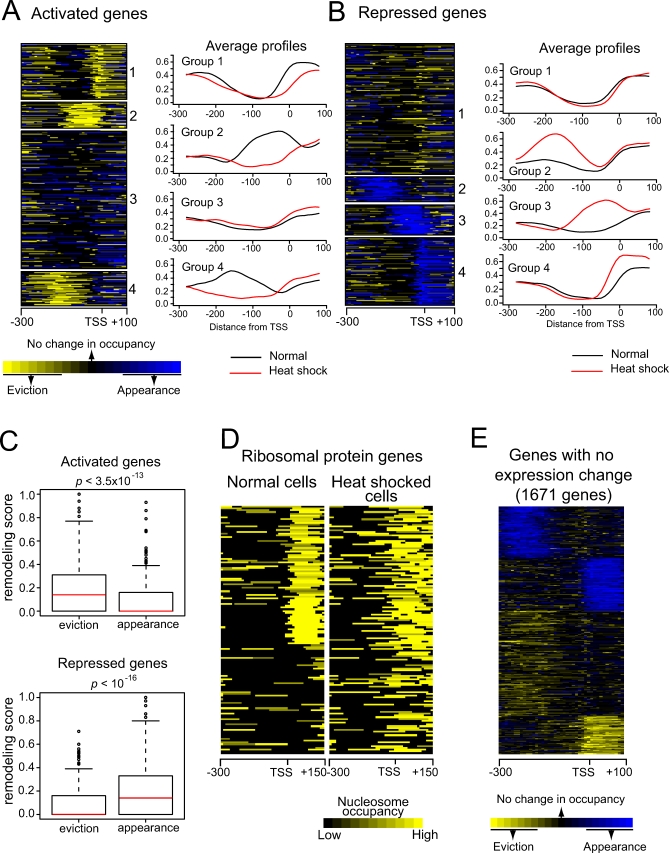
Classification of Promoter Nucleosome Remodeling Profiles All profiles are aligned with respect to the TSS. (A) Remodeling profiles of genes activated greater than 2-fold after heat shock and (B), genes repressed greater than 2-fold by heat shock. Nucleosomes present during normal growth but evicted by heat shock are indicated in yellow, and nucleosomes that appeared after heat shock are shown in blue. The average profiles of nucleosomes in each group before and after heat shock are shown on the right. The *k*-means clustering for (A) and (B) was done based on data from −200 to TSS, but data are shown for −300 to +100. (C) A remodeling score for eviction and for appearance was separately calculated for activated genes and repressed genes (Materials and Methods), and the data were plotted using box plots similar to Figure 3B. Activated genes showed significantly higher eviction scores than appearance scores, whereas repressed genes showed significantly higher appearance scores than eviction scores. (D) Nucleosome positions at the promoters of ribosomal protein genes during normal growth and after heat shock, clustered on data from −200 to +100 bp. (E) Remodeling profiles of genes whose expression changed by less than 1.2-fold after heat shock, clustered based on data from −300 to +100 bp.

We first analyzed remodeling profiles for promoters that were activated at least 2-fold and promoters that were repressed at least 2-fold by heat shock (Figure 4A and 4B). Two well-defined groups (Group 2 and 4) of activated genes contained promoters in which a single nucleosome that covered the promoter during normal growth was evicted upon heat shock, making the promoter more accessible for binding by transcription factors or the general transcription machinery (Figure 4A). Of these, Group 2 showed a significant enrichment for targets of the activator Msn4 (*p* = 0.02) [17] Promoters in Group 1 had a nucleosome-free region between the TSS and −200 bp both before and after heat shock. This group showed a significant enrichment for targets of the transcriptional activator Hsf1 (*p* < 0.02). Group 3 showed enrichment for the remodeler Swi5 (*p* = 0.002).

The difference in nucleosome profiles between Group 1 and Group 2 genes and the differential enrichment of the two major stress transcription factor targets points to two distinct modes of action by these activators. Hsf1 is constitutively bound to many heat shock gene promoters [18]. The nucleosome profiles of Group 1 promoters, which showed enrichment for Hsf1 targets, suggest that Hsf1 binding induces eviction of the nucleosome covering the promoter or precludes its occupancy over this region. On the other hand, Msn4 target promoters (enriched in Group 2) had a nucleosome covering the promoter during normal growth. Our data suggest that translocation of Msn4 into the nucleus upon heat shock [19] and its occupancy of the promoter results in eviction of the nucleosome, and thus facilitates activated transcription.

Genes repressed more than 2-fold after heat shock could also be clustered into four major groups based on their nucleosome remodeling profiles (Figure 4B). Group 2 repressed genes had a nucleosome-free region between −200 and −100 bp upstream of the TSS during normal growth, which was covered by the appearance of a single nucleosome after heat shock. Group 3 repressed genes were characterized by the appearance of a single nucleosome between −125 and +50 bp relative to the TSS after heat shock. Group 1 and Group 4 repressed genes had subtle differences between themselves and between normally growing and heat-shocked cells. They both had a nucleosome-free region between −200 and −100 bp regardless of the transcriptional status of the genes.

The enrichment of transcription factor targets in these four groups based on data from the yeast functional regulatory network [20] and transcription factor ChIP-microarray (ChIP-chip) [17,21] is tabulated in Table S2. Group 3 was significantly enriched for the targets of Rap1, Sfp1, Fhl1, Gcn5, and Esa1, all of which are factors mediating the transcription of ribosomal protein genes during normal growth [22–25]. Consistent with this, ribosomal protein genes were significantly enriched in Group 3 (*p* = 2.6 × 10^−5^). In addition, Group 1 was significantly depleted for targets of all the above-mentioned transcription factors, and was also significantly depleted for ribosomal protein genes (*p* = 6.5 × 10^−4^).

In order to quantitate whether distinct modes of nucleosome remodeling were generally used for gene activation and repression, we calculated a nucleosome remodeling score for both nucleosome eviction and nucleosome appearance (Materials and Methods). Activated genes showed significantly higher nucleosome eviction than nucleosome appearance, whereas repressed genes showed significantly higher nucleosome appearance than eviction (Figure 4C). Although these general trends are expected, we noted that if we clustered remodeling profiles based on more distal promoter regions (−400 to −200 bp upstream of the TSS), we did observe several apparent nucleosome appearance events at activated promoters (Figure S5). At some promoters, nucleosome eviction proximal to the promoter could occur in conjunction with nucleosome appearance more distally, as would be expected for translational repositioning of nucleosomes.

Since ribosomal protein genes form one of the most prominent classes of genes that are transcriptionally repressed by heat shock, we analyzed nucleosome changes at their promoters separately. Ribosomal protein genes were clustered into three classes based on the presence or absence of a well-positioned nucleosome between −50 and +100 bp in normally grown cells, and the nucleosome score. Upon heat shock, we observed the appearance of medium- to high-scoring nucleosomes between −200 and +100 bp of almost all of these ribosomal protein genes in the three groups (Figure 4D).

This analysis of nucleosomal changes at the promoters of the most strongly regulated genes indicates that chromatin remodeling events accompanying transcriptional regulation are restricted to a small number of discrete patterns involving one or two nucleosomes, rather than encompassing a larger domain around the promoter. We also clustered the nucleosome remodeling profiles for genes whose expression did not change appreciably by the physiological perturbation (less than 1.2-fold change). Surprisingly, we still observed similar specific patterns of single-nucleosome remodeling events at many of these promoters, indicating that specific nucleosome events are not universally associated with transcriptional changes (Figure 4E).

### Dynamic Nucleosome Remodeling Causes Changes in the Accessibility of Transcription Factor Binding Sites

**Figure 5:**
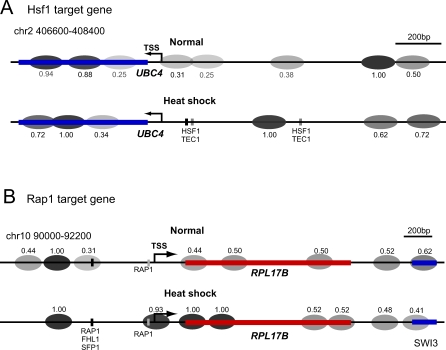
Dynamic Nucleosome Remodeling Affects the Accessibility of Transcription Factor Binding Sites and the TSS (A) Example of nucleosome eviction at the heat-shock–activated *UBC4* promoter (blue line). Nucleosomes defined by our sequencing data are indicated by ovals, colored according to their stability score. The positions of transcription factor binding sites are from [17] and are shaded according to their confidence. Binding sites for other transcription factors are also affected by remodeling (unpublished data), but these are not known to be related to heat shock. (B) Example of nucleosome appearance at the heat-shock–repressed *RPL17B* promoter (red line).

Nucleosome positioning can influence the accessibility of the core promoter as well as binding sites for sequence-specific transcriptional regulators [10,26]. About 90% of the sites occupied by transcription factors on chromosome III under normal growth conditions were depleted of nucleosomes [2]. Examination of single-nucleosome remodeling at promoters that were activated or repressed by heat shock in our data revealed instances where the accessibility of the TSS and of experimentally defined transcription factor binding sites was indeed affected by remodeling. For example, at the *UBC4* promoter, which is activated by heat shock, three moderately positioned nucleosomes covering two distinct Hsf1 binding sites as well as the TSS were evicted, whereas a single, well-positioned nucleosome appeared between the two Hsf1 binding sites (Figure 5A). Conversely, at the *RPL17B* promoter, which is repressed by heat shock, one well-positioned nucleosome appeared after heat shock to cover the TSS and a low-confidence proximal Rap1 binding site. Interestingly, another moderate nucleosome upstream was evicted, exposing a higher confidence distal Rap1 binding site as well as an Fhl1 site (Figure 5B). Such eviction and appearance of nucleosomes at adjacent sites could either reflect translational repositioning or independent events; our experiments cannot distinguish between these two possibilities.

**Figure 6:**
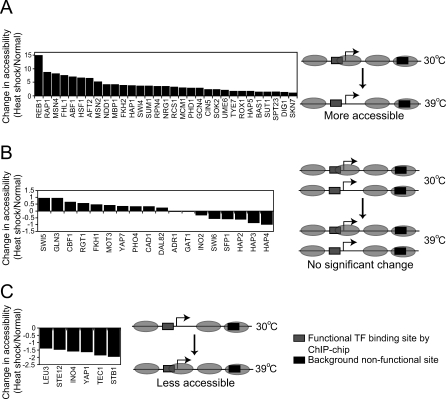
Change in Accessibility of Functional Transcription Factor Binding Sites Transcription factors were classified into three groups based on the change in accessibility of their functional binding sites because of nucleosome repositioning after heat shock. Graphs of accessibility changes in arbitrary units (see Materials and Methods) are plotted for transcription factor binding sites that (A) showed an increase in accessibility, (B) showed no significant change in accessibility, and (C) showed a decrease in accessibility upon heat shock. The right of each graph shows a schematic of the relationship between nucleosomes and transcription factor binding sites.

Based on these observations and other computational predictions of whole-genome nucleosome positions [4], we hypothesized that chromatin remodeling upon transcriptional perturbation could result in changes in the accessibility of the functional binding sites of stress-related transcription factors. To test this hypothesis, we measured the change in accessibility of transcription factor binding sites upon heat shock, by comparing the overlap between functional binding sites for transcription factors measured by ChIP-chip [17] and nucleosome positions before and after heat shock (Figure 6). Of the 101 factors tested, 46 had fewer than 20 functional binding sites each in the genome, and we therefore excluded them from this analysis. The remaining 55 transcription factors could be stratified into three classes based on the change in accessibility of the functional binding sites after heat shock: factors whose binding sites showed an increase in accessibility after heat shock (Figure 6A), factors whose binding sites showed no significant change in accessibility (Figure 6B), and those that showed decreased accessibility after heat shock (Figure 6C). As hypothesized, most of the transcription factors involved in mediating the stress response belonged to the first group. The functional binding sites for several key stress-related transcription factors such as Hsf1, Msn2, Msn4, and Aft2 showed some of the strongest increases in accessibility because of nucleosome repositioning upon heat shock. In addition, binding sites for transcription factors Abf2 and Cbf1, which are involved directly or indirectly in chromatin remodeling [27,28], showed increased accessibility. Surprisingly, we also observed increased accessibility for transcription factors involved in ribosomal protein gene transcription such as Rap1 and Fhl1 (see Figure 5B for an example). These two transcription factors continue to occupy ribosomal gene promoters even during transcriptional repression [29,30], raising the possibility that their occupancy of the promoter under such conditions, facilitated by the increased chromatin accessibility that we observed, could be related to a repressive function. Transcription factors whose binding sites did not show a significant change in accessibility were mainly those involved in the regulation of genes in metabolic pathways.

## Discussion

We have mapped the dynamic remodeling of most nucleosomes in the yeast genome during a transcriptional perturbation using a combination of micrococcal nuclease digestion, isolation of mononucleosome associated DNA and Solexa sequencing. Using a Parzen window–based approach, which is a generally applicable method to analyze all similar datasets derived from ultra-high–throughput sequencing, we defined the dynamic remodeling of approximately 50,000 nucleosomes at single-nucleotide resolution in normally growing cells and in cells that were transcriptionally perturbed by heat shock for 15 min. Our study independently confirms expectations about nucleosomal positioning based on previous smaller scale and lower resolution studies, but also reveals novel features about chromatin structure and transcriptional activity, especially given that previous studies have not examined the dynamic repositioning of nucleosomes in response to genome-wide transcriptional reprogramming.

Our results showed that in addition to a positioned nucleosome at the TSS, genes in general tend to also contain a well-positioned nucleosome at the 3′ end of the coding region. Yeast genes are thus demarcated by a well-positioned nucleosome at each end of their transcribed regions, with a nucleosome-free gap just beyond. This could potentially reflect chromatin organization that facilitates RNA polymerase initiation as well as termination. Most coding regions also showed strongly and regularly positioned nucleosomes, although the strength of the nucleosome positioning was weaker in genes transcribed at high rates. Interestingly, the first well-positioned boundary nucleosome downstream of the TSS, which is likely to be an H2A.Z variant–containing nucleosome based on previous studies [9], showed similar stability in genes transcribed at high and low rates (Figure 3B), suggesting that this chromatin landmark is important for demarcating promoters.

Upon transcriptional perturbation, the majority of nucleosomes did not change positions, either at promoters or within coding sequences (Figures 2 and 3). Gene-specific remodeling was restricted to the discrete eviction, appearance, or repositioning of one or two nucleosomes localized to promoters. Remodeling events at genes that were activated or repressed upon heat shock could be classified into distinct patterns, indicating that there is no simple rule for nucleosome remodeling at promoters to activate and repress genes. Thus, although activation was generally and quantitatively associated with nucleosome eviction and transcriptional repression with nucleosome appearance (Figure 4C), there were cases in which strongly positioned nucleosomes appeared at activated promoters (Figures 5 and S5). Translational repositioning of nucleosomes would seem like eviction and appearance at different spots in the same promoter. These observations suggest that nucleosome remodeling at promoters is not a trivial consequence of transcriptional activity appearing as overall openness of chromatin at activated promoters and obstruction at repressed promoters, but rather, that the precise placement of individual nucleosomes at promoters mechanistically regulates transcription by modulating access of *trans*-acting factors to specific sites.

In addition to chromatin remodeling specifically at regulated promoters, many promoters however showed dynamic single-nucleosome remodeling during the physiological perturbation even in the absence of any resulting transcriptional change (Figure 4E), indicating that selective, activity-specific remodeling was accompanied by a certain number of background, nonspecific remodeling events. We speculate that these background single-nucleosome remodeling events poise promoters for rapid future transcriptional activity, by either assembling partial preinitiation complexes [31], or by exchanging core histones with one or more histone variants [32]. A recent study showed that nucleosomes are globally positioned by Isw2 acting at the boundary between genes and intergenic regions, and that some of the Isw2-dependent remodeling occurs independent of transcription [11]. Therefore, the background remodeling seen in the absence of transcriptional changes in our study could potentially reflect nonspecific remodeling by ISW-like complexes.

We classified transcription factors into three classes based on change in accessibility of their binding sites upon transcriptional perturbation. All the prominent stress-related transcription factors belonged to the category showing a strong increase in accessibility upon transcriptional perturbation. In addition, we found that Rap1 and Fhl1 binding sites showed an increase in accessibility even though the majority of their target genes, namely the ribosomal protein genes, showed a decrease in transcription upon heat-shock stress. When transcription of the ribosomal protein genes is repressed by heat shock, osmotic shock, or inhibition of the TOR pathway by rapamycin, it is known that Ifh1 leaves the promoter, but Rap1 and Fhl1 remain bound [30]. It is possible that Rap1 and Fhl1 play a role in recruiting chromatin remodelers to bring about a repressive chromatin structure at the ribosomal protein genes. Previous studies have indicated that the primary discriminant between a functional and a nonfunctional transcription factor binding site in vivo is the presence of stably positioned nucleosomes covering the latter [4,9]. Our results above indicate that superimposed on this, there is a second mode of regulation at functional binding sites of stress-related transcription factors brought about by a stimulus-dependent remodeling of one or two nucleosomes, making the site more accessible for stable binding of transcription factors. Alternatively, binding of the transcription factor(s) could result in the remodeling of nucleosomes via the help of chromatin remodelers.

The work described here is the first study of genome-wide dynamic nucleosome remodeling events at single-base resolution. More such studies in yeast and higher eukaryotes will shed light on the relationship between epigenetic changes at high resolution and the global regulation of gene expression.

## Materials and Methods

### Preparation of mononucleosomes.

Yeast S288C cultures were grown in rich medium and subjected to 15-min heat shock as described previously [18,33]. At the end of 15 min, control and heat-shocked cells (200 ml each) were treated with formaldehyde to a final concentration of 1% for 30 min. The reaction was stopped by adding glycine to a final concentration of 125 mM, and cells were harvested by centrifugation. Cells were washed 2× in PBS and resuspended in 20 ml of Zymolyase buffer (1 M sorbitol, 50 mM Tris [pH 7.4], and 10 mM β-mercaptoethanol). Cells were spheroplasted by treating with 25 mg of 20T Zymolyase, and incubated for 40 min at 30 °C with shaking at 200 rpm. The remainder of the steps were carried out using a modified protocol described in [2]. Briefly, cells were spun down, washed 1× with 5 ml of Zymolyase buffer, and resuspended in 2 ml of NP buffer (1 M sorbitol, 50 mM NaCl, 10 mM Tris [pH 7.4], 5 mM MgCl_2_, 0.075% NP 40, 1 mM β-mercaptoethanol, and 500 μM spermidine). CaCl_2_ was added to a final concentration of 3 mM, and micrococcal nuclease digestions were carried out at concentrations ranging from 100 U/ml to 600 U/ml for 10 min at 37 °C. The reactions were stopped by adding 100 μl of 5% SDS and 50 mM EDTA. A total of 3 μl of 20 mg/ml proteinase K was added to each tube, and incubated at 65 °C overnight. The DNA was purified by phenol-chloroform-isoamyl alcohol (25:24:1) extraction, and precipitated using ethanol. The DNA was treated with DNase-free RNase, re-extracted with phenol-chloroform-isoamyl alcohol, precipitated with ethanol, and resolved on a 1.25% agarose gel alongside a 100-bp ladder. The mononucleosome size band (approximately 150–200 bp) was excised and purified using the Invitrogen Pure-Link quick gel extraction kit. The purified DNA was sequenced using Solexa sequencing technology.

### RNA isolation and expression profiling.

S288C cells from 50-ml cultures before and after heat shock at 39 °C for 15 min were resuspended in 8 ml of AE buffer (50 mM sodium acetate [pH 5.2], 10 mM EDTA, 1.7% SDS). RNA extraction, cDNA labeling, and microarray manufacture and hybridizations were done as described previously [18,20,33]. For absolute expression analysis, sheared genomic DNA was labeled with Cy3, and cDNA was labeled with Cy5. For relative expression-change analysis, cDNA from heat-shocked cells was labeled with Cy5, and cDNA from normally grown cells was labeled with Cy3. The labeled cDNAs were mixed and hybridized onto DNA microarrays for 12–16 h. The arrays were washed, dried, and scanned with a Axon 4000B scanner (Molecular Devices). Cy5/Cy3 ratios were quantitated using GenePix Pro software and analyzed using Acuity microarray informatics software after filtering to exclude bad spots.

### qPCR validation.

Primer pairs used in Figure 1 were designed to cover three peaks and three troughs in the promoter of *PHO5* just upstream of the known Pho4 binding and DNaseI hypersensitive site [12]. Control primers used for normalization were designed in the region between *YCR023C* and *YCR024C*. qPCR was performed using SYBR green chemistry on an ABI 7900 instrument. Enrichment of target loci in the ChIP sample relative to sonicated genomic DNA was calculated for both unstressed cells and cells subjected to heat shock.

### Nucleosome position detection.

Solexa sequencing reads were mapped back to the Oct 2003 yeast genome assembly obtained from the *Saccharomyces* Genome Database (SGD) (http://www.yeastgenome.org/) and only reads that mapped uniquely to the genome were considered in the majority of our analysis. We generated 514,803 and 1,036,704 uniquely aligning reads for the normal and heat-shock growth conditions, respectively. Reads mapping to the plus and minus strands were processed separately. Reads were clustered using a Parzen window–based approach. Essentially, a Gaussian kernel was centered on each base pair in the genome, and a weighted score was calculated at that position. The mean of the Gaussian was taken as the position under consideration, with the standard deviation (smoothing bandwidth) set at 20 bp. Each read contributed to the mean position based on its kernelized distance from the mean. The weighted score indicated the likelihood of finding an edge of the nucleosome at the position. Thus, the entire genome was converted into a likelihood landscape that was further processed to find local maxima (Figure S6). These maxima were then treated as centers of a cluster. Membership of a read in a cluster was based on its relative contribution to the weighted score of the center. The number of reads assigned to a cluster was defined as the unweighted score of that cluster. We reasoned that a stable nucleosome would be expected to result in a denser clustering of the reads than an unstable one. The denser clustering of the reads results in better concordance of the unweighted score to the weighted score. Hence, each cluster was assigned a stability score that was calculated as the ratio of the unweighted score to the weighted score. Nucleosomes were identified as a plus cluster followed by a minus cluster within 100–200 bp. The nucleosome score was calculated as a sum of the plus and minus cluster unweighted scores. The nucleosome stability score was calculated as a weighted average of the individual stability scores of the participating clusters.

### Overlap between unstressed and heat-shock–stressed cells.

Whole-genome maps for unstressed and stressed cells were filtered to exclude nucleosomes that had a normalized score less than 0.2 (see normalization procedure below). For each nucleosome in unstressed cells, the distance to the nearest nucleosome after heat shock was calculated. These data are reported in Table S1. Similar analysis was used to determine the overlap between nucleosome positions determined in this study and those from previous studies [2,4].

### Random simulations to generate a normalization factor.

Reads equal in number to those we obtained from normal and heat-shocked cells were selected at random from the yeast genome assembly Oct 2003, and peak finding was done as described. This process was iterated 20 times. The average maximum score obtained in the simulations was used as a scaling factor to normalize nucleosome peak scores for cells grown at 30 °C. Normalization was done by dividing nucleosome peak scores by the scaling factor. We then calculated a scaling factor for the heat-shock data by multiplying the scaling factor for the 30 °C data by the ratio of the median peak scores for 39 °C to the peak scores for 30 °C. This was done to correct for differences in sequencing depth for the two samples, thus enabling quantitative comparison of nucleosome profiles across the two conditions.

### Average nucleosome profiles for TATA-containing and TATA-less genes and separation by transcription rates.

The upstream −600 bp to downstream +1,000 bp of each uncharacterized and verified ORF in SGD was binned at 10 bp, and nucleosomes were mapped to each bin. The zero point was the TSS. A nucleosome was said to map to a given bin if it completely overlapped with the 10-bp bin. Each bin was assigned the score of the overlapping nucleosome. In the cases where our algorithm detected overlapping positions for a nucleosome, and more than one nucleosome mapped to a single bin, the bin was assigned the highest score. Genes were separated into TATA-containing or TATA-less [34], and the average nucleosome profiles were generated for each group by averaging the scores for the bin across all the genes (973 and 4,382 promoters, respectively). Genes were similarly separated into the top 500 or bottom 500 with respect to transcription rates [16], and average profiles were plotted for these classes.

### Nucleosome positioning periodicity score and dinucleotide positioning profile.

The NPP score was generated by calculating the similarity of the experimentally derived nucleosome profile over the coding region of every gene to an artificially generated profile where six nucleosomes of score 1.0 were regularly placed with 30-bp linker lengths. In general, genes with well-positioned nucleosome had profiles that were most similar to the synthetic profile and hence, had a high NPP score. The first (+1) nucleosome downstream of the TSS is adjacent to a gap and is likely to be more strongly sequence dependent for positioning than a nucleosome that is flanked by other nucleosomes. We therefore derived AA/TT profiles from the sequence underlying the first nucleosome. To derive high-confidence sequence profiles, we aligned all genes to the first nucleosome as shown in Figure 3A. We selected all +1 nucleosomes with a score ≥0.9 for the input set. Since nucleosomes show a dyad symmetry in terms of positioning over DNA, the reverse complement of each sequence in the input set was also included before calculating the profile. We calculated frequency profiles for the dinucleotides AA and TT, and summed and smoothed them using a 3-bp moving average. This high-confidence AA/TT profile was then correlated with the AA/TT profiles derived from all nucleosomes at the +1, +2, +3, and +4 positions.

### Generation of nucleosome remodeling profiles and remodeling score.

Genes that did not a have 200-bp–long promoter region were excluded for this analysis. For all of the genes that passed this filter, the difference between the nucleosome scores in normally grown cells and cells after heat shock was calculated bin-wise from −400 bp upstream to +200 bp downstream of the start codon. For the plots and clusters shown in Figure 4A and 4B, we then created subsets of these data that included either genes that were activated by at least 2-fold, or genes that were repressed at least 2-fold by heat shock. For the cluster in Figure 4E, we selected remodeling profiles that showed a difference in nucleosome score of at least 0.5 between the two growth conditions at three or more positions in the promoter, and also selected genes whose expression did not change by more than 1.2-fold. To calculate the remodeling score, a seven-bin window, corresponding to a distance of 70 bp (approximately half of a nucleosome), was scanned along each profile, and the individual bin scores were averaged for each window. The maximum window score in the positive direction across the entire profile was assigned as the remodeling score for nucleosome eviction while a similar maximum in the negative direction was assigned as the remodeling score for nucleosome appearance.

### Increase in accessibility of transcription factor binding sites after stress.

Transcription factor motifs were mapped across the entire genome using position-weight matrices derived from [17] using Patser [35] at a *p*-value cutoff of 0.01. These were considered the putative binding sites while the functional (“true”) binding sites were derived from published ChIP-chip data [17,18,36]. A functional motif was considered to be occupied, and therefore not accessible, if it overlapped with a nucleosome that had a score ≥ 0.5. The occupancy of the ChIP-chip binding sites was compared to that of the putative motif binding sites, and a hypergeometric distribution was used to calculate *p*-values. This analysis was done with data from both normal and heat-shock conditions. To calculate the significance of the change in binding site occupancy upon heat shock, the *p*-values for the heat-shock nucleosome data were divided by the *p*-values derived from the normal condition data.

## Supporting Information

Figure S1 Comparison of Nucleosome Positions Before and After Heat Shock, As Well As with Previously Reported Nucleosome Positions (A) and (B) show different regions of the genome. In each track, the raw sequencing data is on top and consists of uniquely aligning reads extended by the average fragment length selected for sequencing. Below this are the nucleosome positions calculated by our analysis algorithm, with their scores shown next to their positions (see Figure S6). Previously reported nucleosome positions as reported by Yuan et al [2] and Lee et al [13] are indicated.
(1.7 MB EPS)

Figure S2 Average Nucleosome Profiles after Heat Shock (A) Nucleosome profile of all genes in the yeast genome from −600 bp to +1,000 bp with respect to the TSS. Nucleosome positions are shown as gray ovals below the profile. The intensity of the filled oval reflects the average probability score of the nucleosome, and the dotted oval around the filled oval marks the spread of that nucleosome across all genes.
(B) The 3′ end of genes is marked by a strongly positioned nucleosome, followed by a relatively nucleosome-free region. The inset shows the 3′ end of convergently transcribed genes in which the 3′ end is not followed by another promoter.
(C) Average nucleosome profiles for TATA-containing (973) and TATA-less (4,382) promoters, aligned with respect to the TSS.
(950 KB EPS)

Figure S3 Internucleosomal Linker Length Distribution in the Yeast Genome Linker lengths were binned into 10-bp (top) or 5-bp bins (bottom), and their frequency distribution was plotted. The most frequent inter-nucleosomal distance, or linker length, was 25–30 bp. The small peak of linker length at 180 bp in the top graph likely reflects the nucleosome-free region at promoters.
(1.5 MB EPS)

Figure S4 TBP Occupancy of Promoters and Absolute Expression Levels of the Different Classes of Genes with Distinct Promoter Nucleosome Profiles Shown in Figure 2C (A) Box plots showing TBP occupancy using data derived from [33], and (B) box plots showing absolute expression levels before and after heat-shock stress. Absolute expression levels were measured as the log_2_ ratio in a DNA+RNA hybridization on genomic microarrays.
(1.8 MB EPS)

Figure S5 *k*-Means Clustering of Nucleosome Remodeling Profiles over Heat-Shock–Activated Promoters from −400 to −200 bp Upstream of the TSS Nucleosome eviction upon heat shock is indicated by yellow, and nucleosome appearance after heat shock is indicated by blue. Clusters 1 and 2 together were significantly enriched for targets of Hsf1 (*p* < 0.04). Although cluster 3 shows nucleosome appearance, this set could include promoters where a nucleosome was evicted from a downstream region and repositioned upstream (e.g., *UBC4* as shown in Figure 5). It could also include promoters where a nucleosome is appearing to cover a repressor site in the heat-shock–activated promoter, or actually appearing at the promoter of another divergently transcribed gene that is repressed by heat shock.
(744 KB EPS)

Figure S6 The 0.5-kb Window Showing Parzen Window–Based Peak Detection (A) Reads mapping to the plus strand (red) and minus strand (blue) were processed separately.
(B) Each base position was assigned a score that was derived from the sum of the relative contributions of all reads in its neighborhood as defined by a Gaussian kernel positioned at that coordinate. A local maximum on the plus strand (red) followed by a corresponding maximum on the minus strand (blue) within a distance of 100 to 200 bp defines a nucleosome. Peaks that were assigned higher Parzen scores defined higher confidence nucleosomes as shown by the grey shading.
(1.4 MB EPS)

Table S1 Nucleosome Overlaps (A) Overlap between nucleosomes mapped in this study with previous studies. Percentages were calculated with reference to the lower of the two numbers considered in the overlap. The threshold for displacement was ≤50 bp.
(B) Overlap between nucleosome positions before and after transcriptional perturbation in this study.
(62 KB DOC)

Table S2 Enrichment and Depletion of Transcription Factor Targets in Nucleosome Profile Clusters from Figure 4B (66 KB DOC)

## Abbreviations

ChIP: chromatin immunoprecipitation
ChIP-chip: chromatin immunoprecipitation–microarray
NPP: nucleosome positioning periodicity
qPCR: quantitative real-time PCR
TSS: transcription start site
