# Supplementary material for: Dynamic Remodeling of Individual Nucleosomes Across a Eukaryotic Genome in Response to Transcriptional Perturbation
Source: PLoS Biol. 2008 Mar 18;6(3):e65. doi: 10.1371/journal.pbio.0060065 (PMC2267817; doi:10.1371/journal.pbio.0060065)
Supplement: Supplementary file 1 [file pbio.0060065.st001.doc]

**Supplementary Table 1:** Nucleosome overlaps. **(A)** Overlap between nucleosomes mapped in this study with previous studies. Percentages were calculated with reference to the lower of the two numbers considered in the overlap. The threshold for displacement was ≤ 50 bp.

|  | Score cut-off | Normally growing cells | After heat shock stress |
| --- | --- | --- | --- |
| Nucleosomes mapped on chromosome III by Yuan et. al. (Science (2005) 309: 626-30) | 0.25 | 75.6% | 79.3% |
|  | 0.5 | 51% | 55% |
| Nucleosome bound sequences isolated by Segal et. al. (Nature (2006) 442: 772-8) | 0.25 | 73.5% | 78.8% |
|  | 0.5 | 51.5% | 60.6% |
| Nucleosomes mapped by Lee et. al. (Nature Genetics (2007) 39: 1235-44) | 0.25 | 79.3% | 78.2% |
|  | 0.5 | 82% | 79.7% |

**(B)** Overlap between nucleosome positions before and after transcriptional perturbation in this study

| Score cut-off | ≤ 20 bp | ≤ 25 bp | ≤ 30 bp | ≤ 50 bp | ≤ 100 bp | > 100 bp |
| --- | --- | --- | --- | --- | --- | --- |
| 0.1 | 52% | 59% | 64% | 77% | 93.3% | 6.7% |
| 0.2 | 56% | 62% | 67% | 79% | 93.8% | 6.2% |
| 0.25 | 54% | 60% | 65% | 76% | 90.4% | 9.6% |
| 0.3 | 55% | 61% | 66% | 77% | 90.1% | 9.9% |
| 0.4 | 55% | 61% | 65% | 75% | 86% | 14% |
