# Supplementary material for: Dynamic Remodeling of Individual Nucleosomes Across a Eukaryotic Genome in Response to Transcriptional Perturbation
Source: PLoS Biol. 2008 Mar 18;6(3):e65. doi: 10.1371/journal.pbio.0060065 (PMC2267817; doi:10.1371/journal.pbio.0060065)
Supplement: Supplementary file 2 [file pbio.0060065.st002.doc]

**Supplementary Table 2:**	Enrichment/depletion of transcription factor targets in nucleosome profile clusters from Fig. 4B

ChIP-chip Targets

|  | **Enriched** | **Depleted** |
| --- | --- | --- |
| **ESA1 (Robert et. al.)** |  |  |
| Group 3 | (p = 0.008) |  |
| Group 1 |  | (p = 8 x 10^-4^) |
| **GCN5 (Robert et. al.)** |  |  |
| Group 3 | (p = 0.01) |  |
| Group 1 |  | (p = 0.007) |
| **FHL1 (Harbison et. al.)** | | |
| Group 3 | (p = 0.004) |  |
| Group 4 | (p = 0.0015) |  |
| Group 1 |  | (p = 1.5 x 10^-4^) |
| **SFP1 (Harbison et. al.)** | | |
| Group 3 | (p = 3.1 x 10^-5^) |  |
| Group 1 |  | (p = 0.005) |
| **RAP1 (Lieb et. al.)** | | |
| Group 3 | (p = 1.2 x 10^-4^) |  |
| Group 1 |  | (p = 1.4 x 10^-4^) |

Yeast functional regulatory network (Hu et. al. Nat Genet, 2007. 39(5): p. 683-7)

|  | **Enriched** | **Depleted** |
| --- | --- | --- |
| **HSF1** | | |
| Group 3 | (p = 1.6 x 10^-4^) |  |
| Group 1 |  | (p = 7.5 x 10^-3^) |
| **CST6** | | |
| Group 3 | (p = 0.002) |  |
| **SPT10** | | |
| Group 3 | (p = 4.8 x 10^-4^) |  |
| Group 1 |  | (p = 0.009) |
| **SFP1** | | |
| Group 3 | (p = 6 x 10^-4^) |  |
| Group 1 |  | (p = 0.01) |
| **RAP1** | | |
| Group 3 | (p = 1.4 x 10^-4^) |  |
| Group 1 |  | (p = 0.015) |
| Group 2 |  | (p = 0.018) |
